# Distinctive *in vitro* ATP Hydrolysis Activity of AtVIPP1, a Chloroplastic ESCRT-III Superfamily Protein in *Arabidopsis*

**DOI:** 10.3389/fpls.2022.949578

**Published:** 2022-07-12

**Authors:** Norikazu Ohnishi, Manabu Sugimoto, Hideki Kondo, Ken-ichi Shioya, Lingang Zhang, Wataru Sakamoto

**Affiliations:** ^1^Institute for Plant Science and Resources, Okayama University, Kurashiki, Japan; ^2^School of Life Sciences, Inner Mongolia University, Hohhot, China

**Keywords:** ATPase, calcium, chloroplast, ESCRT-III superfamily, GTPase, photosynthesis, thylakoid membrane

## Abstract

Vesicle-inducing protein in plastid 1 (VIPP1), characteristic to oxygenic photosynthetic organisms, is a membrane-remodeling factor that forms homo-oligomers and functions in thylakoid membrane formation and maintenance. The cyanobacterial VIPP1 structure revealed a monomeric folding pattern similar to that of endosomal sorting complex required for transport (ESCRT) III. Characteristic to VIPP1, however, is its own GTP and ATP hydrolytic activity without canonical domains. In this study, we found that histidine-tagged *Arabidopsis* VIPP1 (AtVIPP1) hydrolyzed GTP and ATP to produce GDP and ADP *in vitro*, respectively. Unexpectedly, the observed GTPase and ATPase activities were biochemically distinguishable, because the ATPase was optimized for alkaline conditions and dependent on Ca^2+^ as well as Mg^2+^, with a higher affinity for ATP than GTP. We found that a version of AtVIPP1 protein with a mutation in its nucleotide-binding site, as deduced from the cyanobacterial structure, retained its hydrolytic activity, suggesting that *Arabidopsis* and cyanobacterial VIPP1s have different properties. Negative staining particle analysis showed that AtVIPP1 formed particle or rod structures that differed from those of cyanobacteria and *Chlamydomonas*. These results suggested that the nucleotide hydrolytic activity and oligomer formation of VIPP1 are common in photosynthetic organisms, whereas their properties differ among species.

## Introduction

Membrane remodeling is a crucial cellular process that is involved in endocytosis, exocytosis, fission, abscission, and membrane repair. Among these, effective membrane repair is indispensable to cope with damage to cells and organelles, ensuring cell survival in adverse conditions. Chloroplasts, which are vulnerable to light stress due to the photosynthetic reactions taking place within the organelle, are no exception to the requirement for membrane remodeling of both the envelope and thylakoid membranes, yet little is understood regarding the molecules involved in this remodeling. Vesicle-inducing protein in plastid 1 (VIPP1) has been shown to play a pivotal role in thylakoid membrane formation and maintenance. Initially found as localized to both the chloroplast envelope and thylakoid membranes (Li et al., 1994), VIPP1 has been recognized as an essential protein in chloroplasts, and it is highly conserved within oxygenic photosynthetic organisms (Vothknecht et al., 2012; Zhang and Sakamoto, 2013, 2015).

Based on cumulative research spanning more than two decades, multiple roles of VIPP1 related to the organization of chloroplast membranes have been proposed (Kroll et al., 2001; Lo and Theg, 2012; Zhang et al., 2012; Hennig et al., 2015; McDonald et al., 2015), along with the formation of photosynthetic protein supercomplexes (Zhang et al., 2014), through provision of structural lipids (Nordhues et al., 2012) and thylakoid formation via vesicles (Aseeva et al., 2007). The involvement of VIPP1 in protection against stress has also been suggested (Zhang et al., 2016a,b). These studies implied that the important functions of VIPP1 converge around its lipid-binding properties. Indeed, previous *in vitro* studies suggested that VIPP1 induces membrane remodeling, destabilization, and fusion (Hennig et al., 2015; Heidrich et al., 2017; Junglas et al., 2020). Helical structures engulfing liposomes, observed in *Chlamydomonas in vitro*, also support its remodeling activity (Theis et al., 2019). Recent reports on the ring structure of cyanobacterial VIPP1 oligomers, resolved by cryo-electron microscopy (cryo-EM) single particle analysis, demonstrated that its lipid binding is orchestrated by luminal hydrophobic columns derived from the N-terminal α-helix of VIPP1 (Gupta et al., 2021). Likewise, bacterial PspA, which is considered as an ancestral protein of VIPP1, was shown to form helical oligomers (Junglas et al., 2021). In this structure, each monomer forms a hairpin structure similar to not only VIPP1 but also endosomal sorting complex required for transport (ESCRT) III, which is involved in various kinds of membrane remodeling events and also recently recognized as part of the membrane repair machinery (McCullough et al., 2013; Sonder et al., 2019). These findings together demonstrate that VIPP1 plays a fundamental role in chloroplast membrane integrity, similarly to PspA protecting plasma membranes against ion leakage in *Escherichia coli* (Kobayashi et al., 2007).

One unique feature of VIPP1 is its nucleotide-hydrolysis activity (of GTP and ATP), despite the lack of a canonical nucleotide-binding domain. For GTP hydrolysis, we first reported that purified His-tagged protein derived from *Arabidopsis* VIPP1 and *E. coli* PspA had GTPase-like activity *in vitro* (Ohnishi et al., 2018). In general, the contribution of GTPase activity to membrane fusion steps is known, as exemplified by dynamin and its protein family (Ferguson and De Camilli, 2012). GTP hydrolysis activity also appears to contribute to membrane-remodeling in chloroplasts; reportedly, vesicle budding can be prevented by GTPase inhibitors (Westphal et al., 2001), and stromal GTP is required for the integration of light-harvesting complex proteins into thylakoid membranes (Hoffman and Franklin, 1994). Several chloroplast-localized GTPases have been suggested to play roles in membrane dynamics (Lindquist and Aronsson, 2018). Additionally, a recent study showed a facilitative effect of GTP on membrane fusion activity of IM30 (Junglas et al., 2020). These facts imply the membrane remodeling function of VIPP1 through GTPase activity.

For ATP hydrolysis, cyanobacterial and green-algal VIPP1s were recently shown to have both GTPase and ATPase activities (Gupta et al., 2021; Siebenaller et al., 2021). Supporting these findings, a novel nucleotide-binding pocket has been identified in the VIPP1 ring structure that is associated with its oligomeric complex formation. This study by Gupta et al. demonstrated that the NTPase activity of VIPP1 is prerequisite for its full activity (Gupta et al., 2021). ESCRT-III is considered to utilize ATP hydrolysis for disassembling ESCRT proteins after membrane fission (McCullough et al., 2013), which derives from Vps4 ATPase (Mierzwa et al., 2017; McCullough et al., 2018). This ATPase also induces membrane neck constriction and severs membrane tubes (Schöneberg et al., 2018; Maity et al., 2019). Moreover, there are several enzymes that hydrolyze both GTP and ATP (Dutta et al., 2009; Cheung et al., 2016; Unciuleac et al., 2016; Shu et al., 2019). VIPP1 harboring NTPase activity *per se* represents a novel ESCRT-III superfamily protein, although its precise role in membrane remodeling remains unclear.

To investigate the NTPase activity of *Arabidopsis* VIPP1 (AtVIPP1) further, here we assessed the ATP hydrolysis activity. The present data indicated that AtVIPP1 is capable of ATP-binding, ATP-hydrolysis, and ADP production in addition to GTP hydrolysis. Disruption of the putative nucleotide-binding domain deduced from *Synechocystis* VIPP1 did not disturb its activity, raising the possibility that VIPP1 functions differently between cyanobacteria and chloroplasts. The ATP hydrolysis reaction depended on both Mg^2+^ and Ca^2+^ ions as cofactors, unlike the high dependency of the GTP hydrolysis activity only on Mg^2+^. Notably, the *K*_M_ value in the presence of Ca^2+^ was much lower than that in the presence of Mg^2+^, indicating high affinity to ATP. Our results suggested a distinct role of this ATP hydrolysis activity from the GTP hydrolysis activity *in vivo*.

## Materials and Methods

### Preparation of Recombinant His-Tagged Recombinant Proteins

The expression vectors for wild-type and truncated VIPP1 (ΔH1, ΔH5-7) were a kind gift from Dr. Ute Vothknecht (Otters et al., 2013). The expression vector for the N16I mutant protein was prepared based on the vector for wild-type VIPP1 reported previously (Ohnishi et al., 2018). The expression vectors for other mutant proteins (V10E, V11E, R44K, E126Q, E179Q, and E126Q/E179Q) were also prepared based on that for wild-type AtVIPP1 using a mutagenesis kit (PrimeSTAR Mutagenesis Basal kit, TaKaRa Bio, Kusatsu, Japan). The primers used for the mutagenesis are summarized in Supplementary Table 1.

All His-tagged recombinant proteins were prepared as described in Ohnishi et al. (2018) with slight modifications. Overexpression of the proteins was conducted at 27°C in the *E. coli* strain BL21(DE3)pLysS. The cells carrying overexpressed protein were pelleted, frozen in liquid nitrogen, and then stored at −30°C until starting Ni^2+^-affinity purification. The following procedures (Ni^2+^-affinity purification, replacement of buffer, concentration of protein, preparation of glycerol-containing stock solution) were done within a day (typically 10 h) to avoid the loss of ATP/GTP hydrolysis activity. The soluble fraction extracted from *E. coli* cells that had been obtained from ∼25 mL of culture was mixed with 0.65 mL of Ni-NTA agarose (GE Healthcare, Chicago, IL) and then incubated at 4°C for 2.5 h. Based on the elution profiles obtained in the previous study (Ohnishi et al., 2018), the procedures after this step was modified except for purification of the ΔH1 and V11E mutants. After collection of flow through as un-bound fraction, the Ni-NTA column was washed with 12 mL of 150 mM imidazole-containing buffer (150 mM imidazole, 20 mM Tris-HCl [pH 8.0], 500 mM NaCl). Subsequently, proteins bound to the Ni-NTA column were stepwise eluted with 0.5 mL of 1 M and 1.5 mL of 1.5 M imidazole-containing buffer (1 or 1.5 M imidazole, 20 mM Tris-HCl [pH 8.0], 500 mM NaCl). The first and second fractions were used for further preparation. For elution of ΔH1-His and V11E-His, the Ni-NTA column was washed with 12 mL of 25 mM imidazole-containing buffer (25 mM imidazole, 20 mM Tris-HCl [pH 8.0], 500 mM NaCl), then the bound protein was stepwise eluted by 0.5 mL of imidazole-containing buffer (50, 100, 150, 200, and 250 mM imidazole, 20 mM Tris-HCl [pH 8.0], 500 mM NaCl). The fractions of 200-250 mM imidazole elution were collected and used for further preparation (Ohnishi et al., 2018).

After elution, the imidazole-containing buffer of the obtained fractions was exchanged with 2 × VIPP1 storage buffer (100 mM HEPES-NaOH [pH 7.5], 100 mM KCl) using a gel filtration column midiTrap G-25 (GE Healthcare). The obtained fractions containing the desired protein were confirmed by SDS-PAGE and Coomassie brilliant blue (CBB) staining (see below), then subjected to a centrifugation (7,500 × *g* at 4°C) for >2 h with an Amicon Ultra-4 (10K) (Merck KGaA, Darmstadt, Germany) ultrafiltration column to concentrate the recombinant protein. The concentration of protein was estimated using a Bradford Protein Assay Kit (BioRad Laboratories Inc., Hercules, CA). An aliquot of the protein solution (∼10 μL) was diluted to 1.0 μg μL^–1^ with 2 x VIPP1 storage buffer, and then mixed with equal quantity of glycerol to establish a 0.5 μg μL^–1^ of stock solution. The stock solutions were stored at −30°C and used within 1 week for ATP- and GTP-hydrolysis assays. The rest of the concentrated protein solution without glycerol was immediately frozen in liquid nitrogen and was kept at −80°C, and then used for EM analyses.

### ATP- and GTP-Hydrolysis Activity

Measurements of ATP- and GTP-hydrolysis activity were made using an ATPase or GTPase assay kit (Expedeon Ltd., Cambridge, United Kingdom), a dye-based detection system with PiColorLock™, which is described as “superior malachite green reagent highly suppressing non-enzymatic ATP/GTP hydrolysis” in the manufacturer’s instructions. We conducted all reactions at a 200 μL scale in accordance with the instruction manual. The reaction mixture contained 50 mM Tris-HCl (pH 7.5), 2.5 mM MgCl_2_, 0.5 mM ATP or GTP, to which 1.0 μg or 0.5 μg of His-tagged recombinant protein was added for ATP- or GTP-hydrolysis assays, respectively. For the analyses of divalent cation, MgCl_2_ was replaced with other kinds of chloride (CaCl_2_, MnCl_2_ and ZnCl_2_) in the same concentration. The reaction mixtures were incubated at 37°C for 30 min and then immediately cooled in ice water for 1 min. Subsequently, each reaction was terminated by adding the appropriate amount of PiColorLock™ at room temperature. The released Pi was quantified by measuring absorption at 635 nm and comparing with standard solutions. Because the reaction mixture without only recombinant proteins often showed a pale green color, its absorption was subtracted as the background in each experiment. Unless specifically described, the composition of the reaction mixture and the incubation time were used as standard conditions.

### Dot Blot Assay

Interactions between AtVIPP1-His and ATP were assessed using a dot blot assay as described previously (Diekmann and Hall, 1995) with some modifications. In brief, 5 μg of AtVIPP1wt, ΔH1, or BSA was spotted in a volume of 5 μL onto nitrocellulose membranes. The membrane was then air dried for 20 min at room temperature. The membranes were incubated in the blotting buffer (100 mM Tris-HCl [pH 7.5], 1 mM MgCl_2_, 25 mM KCl, 100 mM NaCl) for at least 1 h at room temperature. After supplementation with 20,000 μCi of [α-^32^P]ATP (specific activity 3000 Ci mmol^–1^), incubation was continued at room temperature for 1 h. The membranes were washed with washing buffer (blotting buffer supplemented with 0.2% Tween-20) three times and were then air dried. The signals were detected using a bio-imaging analyzer (BAS1000; Fuji Photo Film, Tokyo, Japan).

### SDS-PAGE and Immunoblotting Analysis

Aliquots of either soluble proteins obtained from *E. coli* cells or purified recombinant proteins were solubilized by incubation at 75°C for 5 min in the presence of 2% SDS and 0.1 M DTT. The protein samples were centrifuged for 2 min at >20,000 × *g* and then subjected to SDS-PAGE using 12.5% (w/v) polyacrylamide gels. The proteins in the gel were subsequently visualized by staining with CBB Stain ONE (Nacalai Tesque Inc., Kyoto, Japan). In the case of immunoblotting analyses, the separated proteins were blotted electrophoretically onto polyvinylidene fluoride (PVDF) membranes and were probed with polyclonal antibodies raised against AtVIPP1-His. Signals were visualized using a chemiluminescence reagent (Luminata Crescendo Western HRP substrate; Merk KGaA) and detected using a ChemiDoc analyzer (BioRad Laboratories Inc.).

### High Performance Liquid Chromatography Analyses

The reaction mixtures from ATP/GTP hydrolysis for high performance liquid chromatography (HPLC) analyses were prepared with an increased quantity of VIPP1-His (2.5 μg/200 μL reaction) to obtain strong signals at each time point. After incubation at 37°C as described above, the reaction mixtures were kept on ice-water for 0.5–4 h until the HPLC analyses were performed.

An aliquot of the reaction mixture (20 μL) was subjected to HPLC using a Cosmosil C_18_ column (4.6 × 250 mm, Nacalai Tesque) equilibrated with 50 mM potassium dihydrogenphosphate (pH 4.6), 25 mM tetrabutylammonium hydrogensulfate, and 0.5% acetonitrile at a flow rate of 1 mL/min (Akhova and Tkachenko, 2019). The substrate and reaction product were detected using absorption at 254 nm. The concentration of ADP and GDP at each time point was calculated based on the area of standard samples. In order to estimate actual increases, the concentration at the time 0 was subtracted from the values of each time point as the background.

### Sucrose Density Gradient Centrifugation

After an aliquot of solution containing 2 μg of recombinant proteins was filled up to 100 μL with storage buffer (50 mM HEPES-NaOH [pH 7.5], 50 mM KCl), it was overlaid onto a continuous sucrose density gradient (0.4–1.6 M) containing 50 mM HEPES-NaOH (pH 7.5) and 50 mM KCl. Each gradient was centrifuged at 85,000 × *g* for 15 h at 4°C (SW50.1 rotor; Beckman Coulter Inc., Brea, CA). After centrifugation, the gradients were fractionated into 25 fractions (200 μL each) from top to bottom. An aliquot of each fraction was subjected to SDS-PAGE and subsequent immunoblotting analyses with a specific antibody against VIPP1 as described above.

### Negative Staining Electron Microscopy

The protocol used for the EM observations was based on that of Kondo et al. (2009) with minor modifications. Collodion-coated, carbon-stabilized copper grids (400 mesh) were hydrophilized using an EM hydrophilization system (DII-29020HD, JEOL, Ltd., Tokyo, Japan), and approximately 10 μL of AtVIPP1-His protein samples (3.5–5.5 μg/μL) were placed on the grid. Subsequently, the samples on the grids were negatively stained with 2% (w/v) uranyl acetate or an EM stain (EM stainer, Nissin EM Co., Tokyo, Japan) (Nakakoshi et al., 2011). Stained samples were then observed using EM (Hitachi model H-7650 transmission electron microscope, Tokyo, Japan) and photographed as digital images.

## Results

### Evaluation of ATP Hydrolysis Activity of Vesicle-Inducing Protein in Plastid 1-His Protein

**FIGURE 1:**
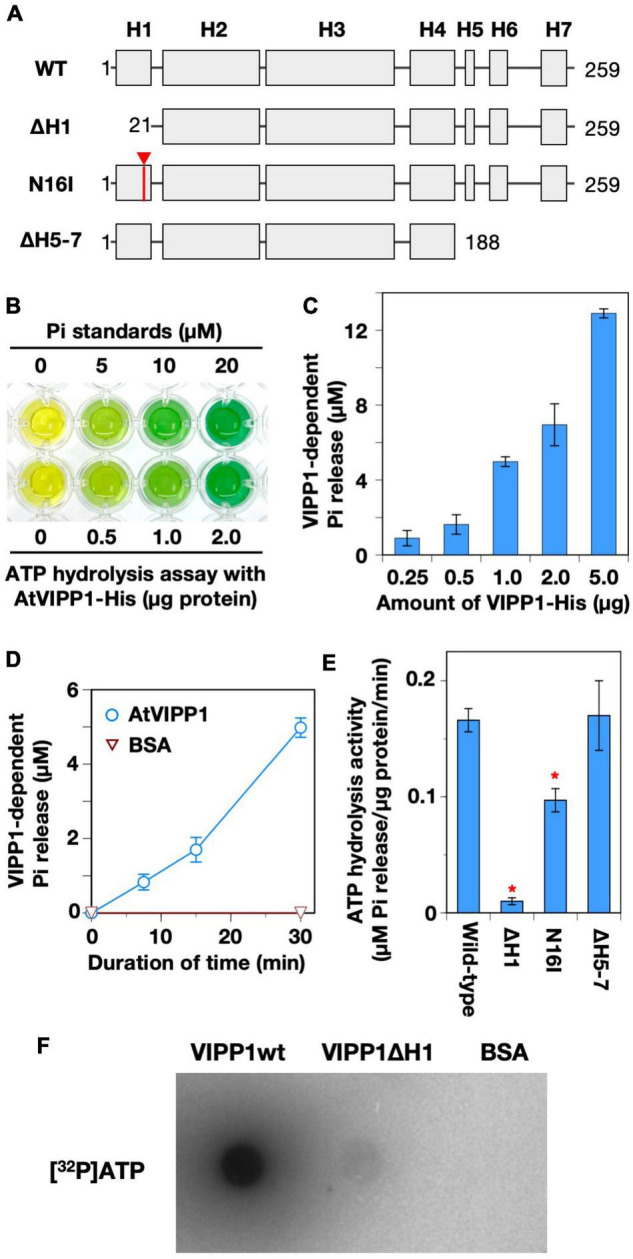
ATP hydrolysis and binding by recombinant AtVIPP1 proteins. **(A)** Schematic illustration of the secondary structure of *Arabidopsis* VIPP1 (adopted from Ohnishi et al. (2018)). H indicates a predicted α-helical structure. The numbers indicate the positions of first (left) to last (right) amino acid of the proteins. The mutation site of N16I is approximately indicated by an arrowhead. The detailed sequence of H1 is shown in Supplementary Figure 4. **(B)** A photograph of typical a malachite green-based assay for ATP hydrolysis. Green color indicates the presence of free inorganic phosphate (Pi). **(C)** The level of Pi released from ATP was analyzed using various amounts of wild-type VIPP1 protein (0.25–5.0 μg in 200 μL reaction mixture) by the malachite green-based assay. **(D)** Time-dependent increase in released Pi in the presence of VIPP1-His protein. The reaction mixtures were incubated for 0, 7.5, 15, and 30 min at 37°C, then free Pi was quantified. Each data point and error bar represents the means and SE, respectively, which were calculated from the results from 4–6 independent experiments. **(E)** The ATP hydrolysis activity of wild-type, N-terminally, and C-terminally mutated VIPP1-His proteins was analyzed. All the analyses were carried out in standard condition (see Methods). Each bar graph and error bar represents the means and SE, respectively, of results from 4–6 independent experiments. Asterisks denote significant differences in the activity from wild-type proteins (Welch’s *t*-test with *p* < 0.01). **(F)** Direct ATP-binding test of VIPP1-His protein. VIPP1-His, ΔH1, and BSA were dotted on a nitrocellulose membrane and then incubated with radio-labeled ATP ([α−^32^P]ATP) at room temperature for 1 h. The signals were detected using a BAS1000 system.

We prepared C-terminally His-tagged VIPP1 proteins (AtVIPP1-His) without its N-terminal transit peptide as used previously (Figure 1A and Supplementary Figure 1A; Ohnishi et al., 2018). First, we tested a Malachite green-based colorimetric detection system as described in the Methods. The increase in released inorganic phosphate (Pi), proportionally to the amounts of AtVIPP-His (within 5 μg range), was readily detected (Figures 1B,C). We also tested other nucleotide triphosphates, CTP and UTP, as substrates. Even in the presence of 2.0 μg of AtVIPP1, the release of Pi was very low, indicating the substrate specificity of AtVIPP1 to only GTP and ATP (Supplementary Figure 2). We selected 1.0 μg of VIPP1-His protein for further analyses. VIPP1-dependent release of Pi from ATP was next examined using a time course experiment. During incubation for 30 min, the amount of Pi increased in a time-dependent manner and reached about 5 μM in the presence of 1.0 μg of VIPP1-His, whereas BSA used as a negative control did not increase the level of Pi in any of the reaction mixtures (Figure 1D).

Vesicle-inducing protein in plastid 1 was originally predicted to form seven α-helices (H1, H2, H3, H4/H5, H6, and H7, Figure 1A), in which the N-terminal H1 is important for lipid-binding (Otters et al., 2013) and H7 forms an intrinsically disordered region (Zhang et al., 2016a). To test the region important for this ATP hydrolysis activity, we prepared N-terminal helix-truncated (ΔH1), H1 point mutated (N16I, also see Supplementary Figures 5A,B), and C-terminal-truncated (ΔH5-7) AtVIPP1s (Ohnishi et al., 2018). As shown in Figure 1E, the activity of ΔH1 was almost abolished, and N16I showed approximately 50% activity compared with wild-type protein. In contrast, the activity of ΔH5-7 was as high as wild-type, although the results were variable. Overall, the trends were similar to those in GTP hydrolysis activity (Ohnishi et al., 2018). The nucleotide-binding assay using radio-labeled [^32^P] ATP showed that AtVIPP1 bound ATP strongly, whereas ΔH1 showed a faint signal (Figure 1F). We concluded that H1 is crucial in both ATP and GTP hydrolysis activities of AtVIPP1.

### High-Performance Liquid Chromatography Detection of ADP/GDP in Nucleotide Hydrolysis Reactions

**FIGURE 2:**
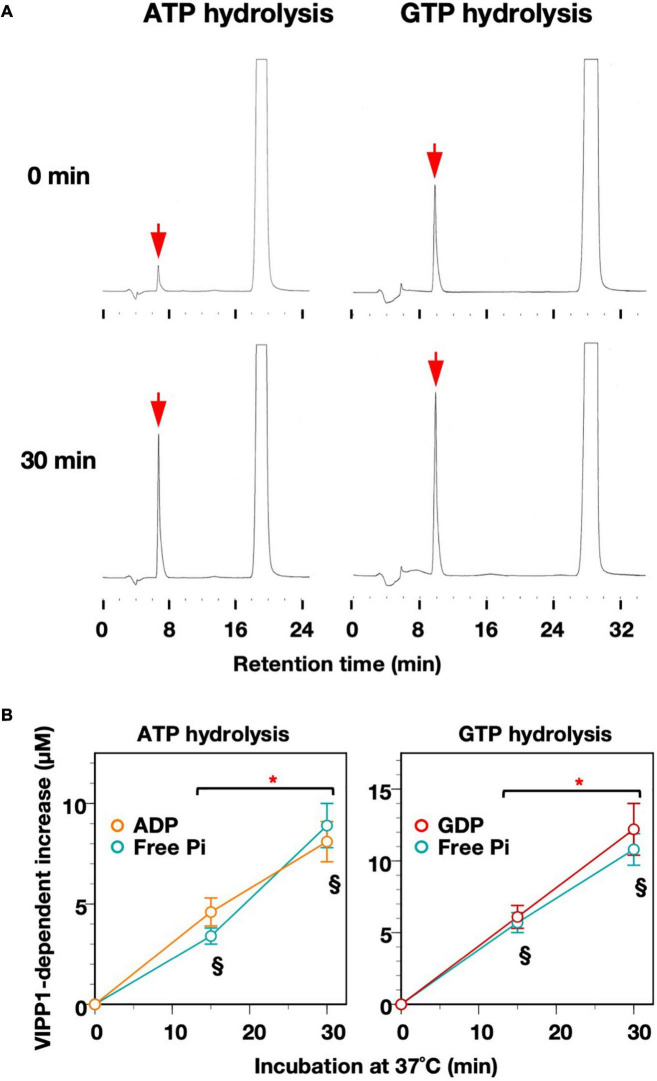
Time-dependent increase in ADP/GDP and Pi. **(A)** Representative elution profiles obtained from the reaction mixtures after ATP- and GTP-hydrolysis. To obtain high enough signals to detect, the reactions were carried out with the addition of 2.5 μg VIPP1-His protein instead of 1.0 μg protein in standard conditions. The elution peaks corresponding to ADP (column for ATP hydrolysis) and GDP (column for GTP hydrolysis) are indicated with red arrows. **(B)** Time course graphs indicating increases in the levels of ADP and GDP, which were obtained by quantification of the results from HPLC analyses in panel **(A)**. The increase in level of Pi analyzed in the same conditions (using 2.5 μg VIPP1-His protein) is also shown in the graphs. Asterisks indicate significant differences between the two time points according to a statistical analysis. § Under the data points denotes no significant difference between the concentration of ADP/GDP and that of free Pi at each time point (Welch’s *t*-test, *p* < 0.05). Error bars: ± SE.

We next assessed what kinds of end product(s) are actually obtained by the hydrolysis reactions: in general, ATPases and GTPases release Pi plus ADP and GDP, respectively, but the enzymes exist that are capable of catalyzing both ATP-to-ADP and ADP-to-AMP (Smith et al., 2002), or GTP-to-GDP and GDP-to-GMP conversions (Schwemmle and Staeheli, 1994; Hyun et al., 2000). HPLC analysis indicated only one elution peak during the reaction except for an over-shoot peak corresponding to the substrate ATP (∼19 min) or GTP (∼27 min). Based on the elution profiles of standard samples (Supplementary Figure 3A), elution peaks that appeared at ∼7 min and ∼9 min were identified as ADP and GDP, respectively (Figure 2A). According to the estimation, ADP increased to 4.6 ± 0.7 and 8.1 ± 1.0 μM at 15 and 30 min, respectively. In the same reaction, Pi calculated by colorimetric detection increased to 3.4 ± 0.4 and 8.9 ± 1.1 μM at 15 and 30 min, respectively (Figure 2B). Statistical analyses showed that, at both time points, there was no significant difference in the concentrations between the level of ADP and that of Pi, indicating a stoichiometric increase in the two products. Similarly, GDP and free Pi were stoichiometric when the reaction was performed in the presence of GTP (Figure 2B). The ΔH1 mutant protein did not increase the level of either ADP or GDP (Supplementary Figure 3B). Based on these results, we concluded that AtVIPP1 catalyzed conversion of ATP to ADP, and of GTP to GDP as well, but neither ADP nor GDP was converted to nucleotide monophosphates, at least in this time scale.

### Preference of pH in the Nucleotide Hydrolysis Reactions

**FIGURE 3:**
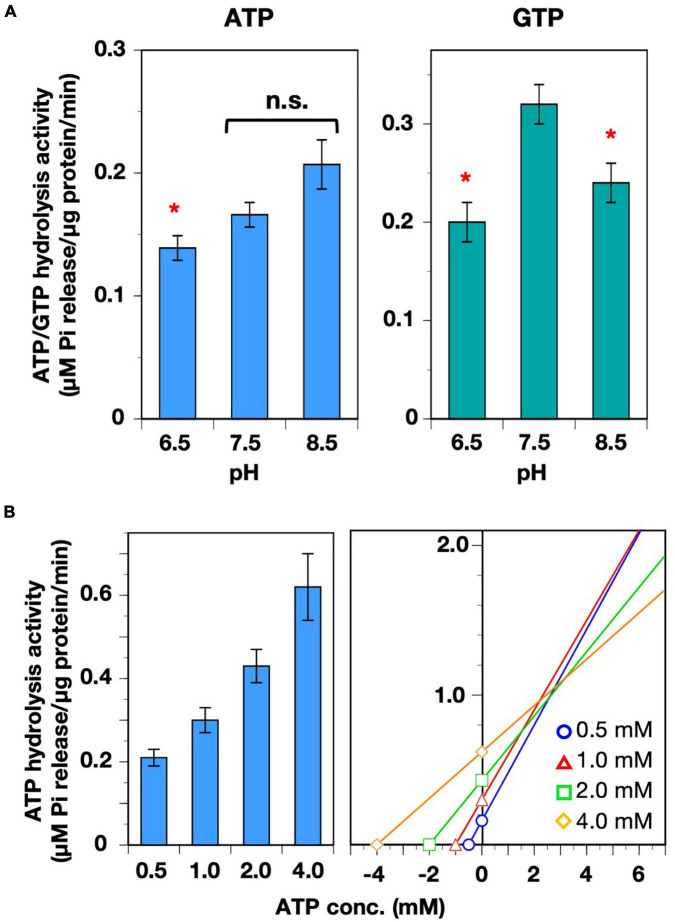
Characterization of ATP hydrolysis activity. **(A)** The pH preferences of both ATP- and GTP-hydrolysis activities were analyzed. Asterisks denote significant differences in the activity from those at pH 7.5 (Welch’s t-test with *p* < 0.01). **(B)** Analyses of *V*_max_ and *K*_M_ of the ATP hydrolysis activity. ATP hydrolysis activity was measured in the presence four ATP concentrations (0.5–4.0 mM). Each bar graph and error bar represents the means and SD, respectively, of results from 5–9 independent experiments (left graph). The concentration of ATP and the average of VIPP1-dependent Pi release are plotted on the graph in accordance with the Cornish–Bowden plot method. The *X* and *Y* values of the intersection points represent *K*_M_ and *V*_max_, respectively (right graph).

We next examined whether the ATP/GTP hydrolysis activity has preference for either acidic, neutral, or alkaline conditions, because each soluble compartment of the chloroplast (*i.e.*, intermembrane space of the envelope, stroma, and thylakoid lumen) possesses different pH values (Höhner et al., 2016), which is subjected to dynamic change during photosynthetic reactions, especially under natural fluctuating sunlight. Among the examined pH conditions, ATP hydrolysis activity was slightly lower at pH 6.5. The activity tended to increase in alkaline conditions, but statistical analyses showed that there was no significant difference in activity between pH 7.5 and pH 8.5. On the other hand, the GTP hydrolysis activity was higher at pH 7.5 than at pH 6.5 or 8.5 (Figure 3A), indicating that neutral conditions were optimal for GTP hydrolysis reactions of AtVIPP1. Because the theoretical *K*_M_ and *V*_max_ values for GTP hydrolysis have been analyzed at pH 7.5 previously, those values for the ATP hydrolysis reaction were also tested (Table 1). We measured ATP hydrolysis activity in the presence of several concentrations of ATP and generated a Cornish–Bowden plot and obtained the *V*_max_ and *K*_M_ values from the average of x and y values of the intersection points (Eisenthal and Cornish-Bowden, 1974; Berges et al., 1994). *V*_max_ and *K*_M_ were calculated as 1.2 μM Pi release/μg protein/min and 3.21 mM, respectively (Figure 3B and Table 1). This indicated that both the catalytic rate and the substrate affinity were lower than those for the GTP hydrolysis reaction we reported previously (Table 1; Ohnishi et al., 2018).

**TABLE 1:** *V*_max_ and *K*_M_ of the ATP- and GTP-hydrolysis activities of AtVIPP1-His.

Substrate	Divalent cation	pH	*V*_max_ (μmol Pi/ μg VIPP1/min)	*K*_M_ (mM)	Reference
GTP	Mg^2+^	7.5	1.9–2.0	2.2	Ohnishi et al., 2018
ATP	Mg^2+^	7.5	1.2	3.21	This work
	Ca^2+^	7.5	0.39	1.07	This work
	Ca^2+^	8.5	0.35	0.22	This work

*AtVIPP1, Arabidopsis vesicle-inducing protein in plastid 1 Pi, inorganic phosphate.*

### Requirement for Different Divalent Cations in the Nucleotide Hydrolysis Reactions

**FIGURE 4:**
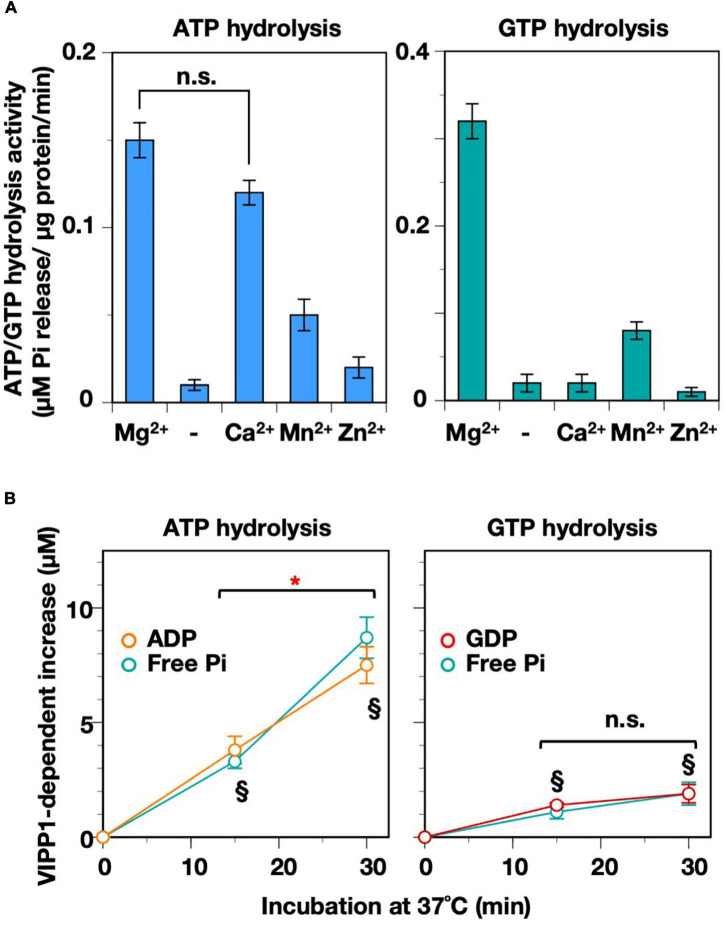
Requirement for divalent cations. **(A)** Both ATP- and GTP-hydrolysis activities were measured in the absence (shown as “–”) or presence of four species of divalent cation (Mg^2+^, Ca^2+^, Mn^2+^, and Zn^2+^). n.s.: No significant differences according to a statistical analysis (Welch’s *t*-test, *p* < 0.01). **(B)** Time course graphs indicating the increase in ADP and GDP in the presence of Ca^2+^, which were obtained from HPLC analyses on Supplementary Figure 4. The level of Pi analyzed in the same conditions (with 2.5 μg VIPP1-His protein) is also plotted on the graphs. An asterisk indicates a significant difference between the two time points, whereas there was no significant difference at the time points shown as “n.s.” according to a statistical analysis. § Denotes that there was no significant difference between the levels of ADP/GDP and that of free Pi at each time point (Welch’s *t*-test, *p* < 0.01, *n* = 4-6).

We showed previously that the GTP hydrolysis reaction of AtVIPP1 is dependent on Mg^2+^ ions (Ohnishi et al., 2018), whereas *Synechocystis* VIPP1 (SynVIPP1) does not require divalent cations (Junglas et al., 2020). In the present study, we tested the effects of four different divalent cations (Mg^2+^, Mn^2+^, Ca^2+^, and Zn^2+^) on both ATP- and GTP hydrolysis activities (Figure 4A). GTP hydrolysis activity was previously analyzed in the presence of Mg^2+^ and Ca^2+^. Although we tested two additional divalent cations, Mn^2+^ and Zn^2+^, neither provided full GTP hydrolysis activity, indicating high dependency on Mg^2+^ ions. In contrast, ATP hydrolysis in the presence of Ca^2+^ was equivalent to that in the presence of Mg^2+^. The products of reactions in the presence of Ca^2+^ were also analyzed by HPLC, showing elution profiles similar to those in the case of Mg^2+^ (Supplementary Figure 4). The concentration of ADP did not differ significantly from Pi at each time point (Figure 4B). These results revealed that the ATP hydrolysis activity was able to function in the presence of either Mg^2+^ or Ca^2+^, unlike the GTP hydrolysis activity.

**FIGURE 5:**
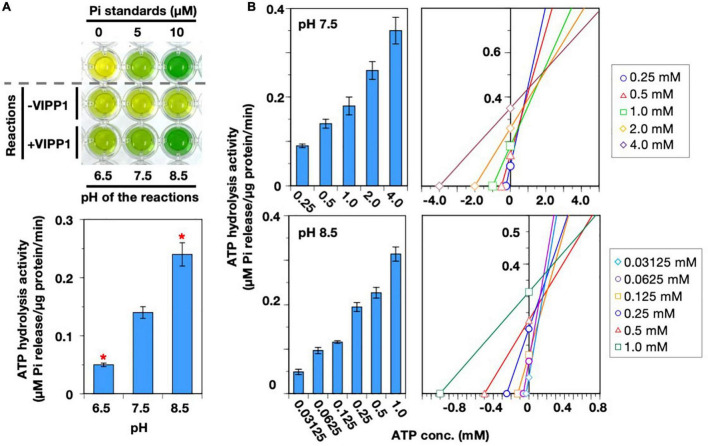
Properties of the Ca^2+^-dependent ATP hydrolysis. **(A)** A photograph of typical results of a malachite green-based assay (upper panel) and the quantified activity of ATP hydrolysis (lower panel) in the presence of Ca^2+^. The reactions were carried out at pH levels (6.5, 7.5, and 8.5). Asterisks in the bar graph denote significant differences from the activity at pH 7.5 (Welch’s *t*-test with *p* < 0.01). **(B)** Analyses for *V*_max_ and *K*_M_ for ATP hydrolysis activity in the presence of Ca^2+^ at pH 7.5 and 8.5. ATP hydrolysis activity was measured in the presence of various ATP concentrations, which are indicated at the bottom of the bar graphs. Each bar graph and error bar represents the means and SD, respectively, of results from 5–9 independent experiments (left graphs). The concentration of ATP and the average of VIPP1-dependent Pi release are plotted on the graph in accordance with the Cornish–Bowden plot method. The *X* and *Y* values of the intersection point represent *K*_M_ and *V*_max_, respectively (right graphs).

We examined the mutual influence of two factors, Ca^2+^ and pH, on the ATP hydrolysis activity. Ca^2+^ ions move across the chloroplast membranes in response to not only light–dark transition (Johnson et al., 1995; Sai and Johnson, 2002) but also abiotic stresses (Sello et al., 2018). We reasoned that the function of AtVIPP1 *in vivo* might be controlled by Ca^2+^, whose flux is a potential candidate monitoring the physiological status of chloroplast membranes in stressed conditions (Zhang et al., 2012, 2016a; Sello et al., 2018). On the other hand, the chloroplast soluble compartments undergo pH changes during photosynthesis as described above. As shown in Figure 5A, alkaline pH seems to give preferable conditions for the Ca^2+^-dependent ATPase activity, unlike the case in the presence of Mg^2+^. At pH 6.5, the activity was less than 50% of that at pH 7.5, whereas pH 8.5 provided high activity, which was almost twice of that at pH 7.5 (Figure 5A, lower panel). We calculated *K*_M_ and *V*_max_ values for the two pH conditions, 7.5 and 8.5 (Figure 5B and Table 1). At pH 7.5, *K*_M_ and *V*_max_ were 1.07 mM and 0.39 μM Pi release/μg protein/min, respectively. Compared with those values in the presence of Mg^2+^, both *K*_M_ and *V*_max_ were low. The values showed a similar tendency at pH 8.5 (*K*_M_: 0.22 mM, *V*_max_: 0.35 μM Pi release/μg protein/min), indicating obviously high affinity for ATP. The low *V*_max_ may be because of the high affinity causing substrate inhibition.

### Effects of Amino Acid Substitution on the ATP/GTP Hydrolysis Activity

**FIGURE 6:**
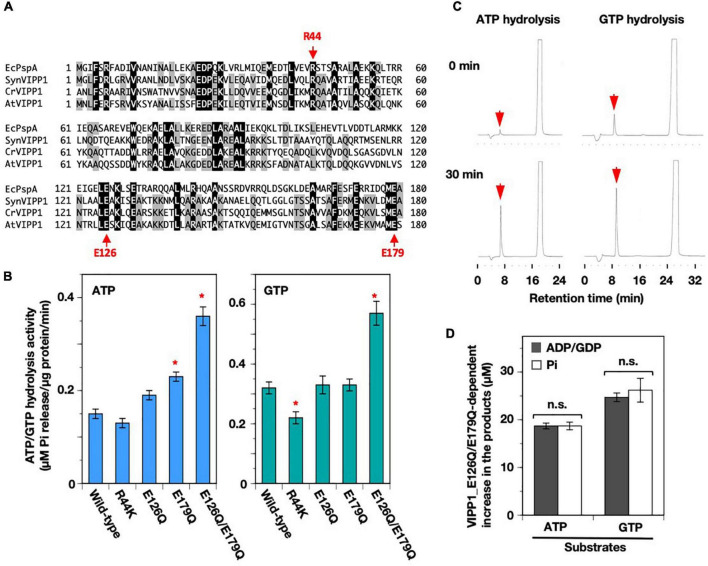
Mutations in the nucleotide-binding pocket predicted from the structure of SynVIPP1. **(A)** Multiple alignment of partial amino acid sequence (1–180) among a PspA (*Escherichia coli*) and three VIPP1s (*Synechocystis*, *Chlamydomonas*, and *Arabidopsis*). The amino acids that contribute to ATP/GTP-binding in cyanobacterial VIPP1 are shown with arrows (R44, E126, and E179). **(B)** ATP- and GTP-hydrolysis activities of wild-type and four point-mutated proteins were analyzed based on *Synechocystis* VIPP1 previously. Asterisks in the bar graphs denote significant differences in activity from the wild-type protein (Welch’s t-test with *p* < 0.01). **(C)** Representative elution profiles of ADP and GDP obtained from the reactions of E126Q/E179Q mutant protein. **(D)** The level of major products obtained in 30-min hydrolysis reactions. There were no significant differences between the values of graphs, which is shown as “n.s.” according to a statistical analysis (Welch’s *t*-test, *p* < 0.01). Error bars: ± SE.

Recently, the detailed structure of cyanobacterial VIPP1 oligomers revealed a novel ATP/GTP-binding pocket, which consisted of three monomers of SynVIPP1. Among the point mutations introduced according to the structure, three of them (R44K, E126Q, and E179Q) reduced the ATP/GTP hydrolysis activity, and the double mutation (E126Q/E179Q) not only abolished the activity but also disrupted its oligomer formation (Gupta et al., 2021). Multiple alignment with PspA from *Escherichia coli* and three VIPP1 proteins from *Synechocystis* sp. PCC6803, *Chlamydomonas reinhardtii*, and *Arabidopsis thaliana*, showed that the three amino acids, R44, E126, and E179, were all conserved (Figure 6A). We introduced these identical point mutations into AtVIPP1 (Gupta et al., 2021). Contrary to our expectations, only the R44K mutant protein showed a slight decrease in GTP hydrolysis activity. E126Q and E179Q had the similar activity to the wild-type protein. Surprisingly, the E126Q/E179Q double mutant protein showed rather high activity (Figure 6B). Consistently, HPLC analysis indicated that only a peak corresponding to ADP or GDP was detected (Figure 6C), in which the concentration of released Pi was comparable to that of ADP and GDP (Figure 6D). These results indicated that the high ATP/GTP hydrolysis activity in Figure 6B was attributed to an accelerated catalysis of ATP to ADP/GTP to GDP, but not to other reactions such as conversion from nucleotide diphosphate to nucleotide monophosphate.

We also tested two kinds of point mutation on H1, V10E, and V11E, each of which reduced the ATP/GTP hydrolysis activity of SynVIPP1 (Gupta et al., 2021). These two residues are conserved not only between SynVIPP1 and AtVIPP1 but also among other species (Ohnishi et al., 2018), and located at the hydrophobic surface of this characteristic amphipathic helix (Zhang and Sakamoto, 2015) (Supplementary Figures 5A,B). We prepared these mutant proteins and analyzed their nucleotide hydrolysis activity (Supplementary Figures 5C,D). V11E had reduced activity, which was similar to the case of SynVIPP1, whereas the activity of V10E was rather higher than that of wild-type protein. These results indicate that, despite of conservation between SynVIPP1 and AtVIPP1, effect of substitution of amino acid residues on nucleotide hydrolysis could be dependent on species.

### Oligomeric Properties of Wild-Type and Mutated AtVIPP1s

To obtain insights about the relationship between nucleotide hydrolysis and oligomer formation, we first carried out sucrose density gradient centrifugation. The majority of wild-type AtVIPP1 was detected in fraction 20, with additional weak signals in fractions 22 and 24 (Supplementary Figure 6). The E126Q/E179Q double mutant also migrated to the same fractions as wild-type protein. However, the signals in fractions 22–24 appeared to be stronger than those for the wild-type, protein, suggesting that a proportion of E126Q/E179Q could form much larger oligomers than the wild-type. In contrast, the V11E mutant was mainly found in fractions 2–4 (Supplementary Figure 6), indicating that its density was far lower than that of the wild-type. These results resembled those of ΔH1, although the nucleotide hydrolysis activity of V11E was rather similar to the N16I mutant, which retained slightly modified high-ordered oligomers (Ohnishi et al., 2018). V11E was also weakly detected around fractions 20–24, indicating the presence of a minor fraction of wild-type-like oligomer.

**FIGURE 7:**
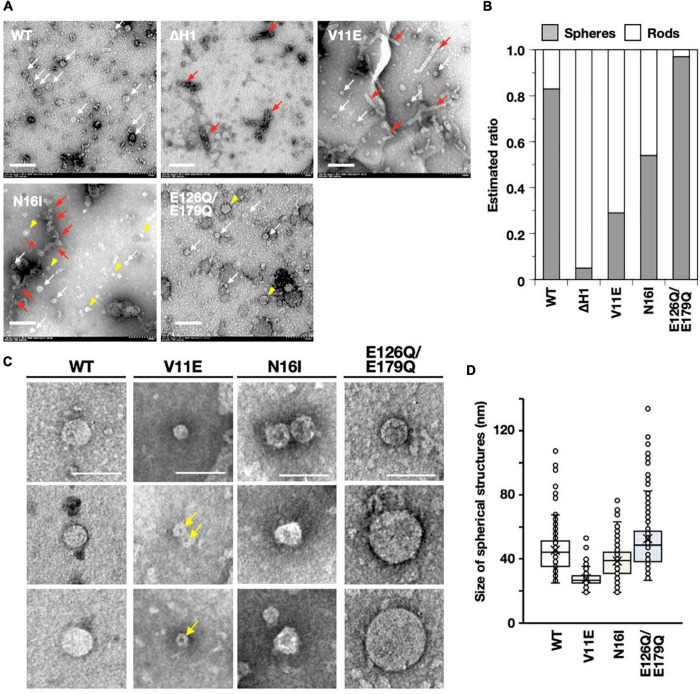
Analyses based on electron micrographs of negative-stained AtVIPP1-His proteins. **(A)** Typical electron micrographs of wild-type, ΔH1, V11E, N16I, and E126Q/E179Q recombinant proteins. White and red arrows indicate spherical and rod-like structures, respectively. Yellow arrowheads on photos of N16I and E126Q/E179Q point to spherical structures that are characteristic in each species. Scale bars represent 200 nm. **(B)** The ratio of spheres to rods in each protein preparation was roughly estimated in accordance with the method in Gupta et al. (2021). The details are shown in Supplementary Figure 8A. **(C)** Examples of spherical structures observed in wild-type, V11E, N16I, and E126Q/E179Q protein preparations. For the three mutants, the upper photos show wild-type-like structures, and the lower two photos represent the structures specifically observed in each mutant. Yellow arrows indicate hole-like shadows located in the center of V11E particles. Scale bars represent 100 nm. **(D)** Distribution of the size of spherical structures in wild-type, V11E, N16I, and E126Q/E179Q protein preparations. The diameters of 100–280 spherical structures were measured manually, as shown in Supplementary Figure 8B, and plotted on box plot graphs. The distribution of every species of protein differed from each other with *p* values of <0.001, which was calculated using the statistical Brunner–Munzel test.

The VIPP1 oligomers from *Synechocystis* (Fuhrmann et al., 2009) and *Chlamydomonas* (Gao et al., 2015; Theis et al., 2019) frequently contained both ring- and rod-like structures. However, the major structure of AtVIPP1 seemed to be spherical particles without a central hole, when observed using negative staining and EM (Otters et al., 2013; Zhang et al., 2016a). As expected, wild-type VIPP1 mostly showed a spherical structure, with minor rod-like structures (Figure 7A and Supplementary Figure 7). The diameter of the spherical oligomers ranged from 30 to 50 nm, with some much smaller and larger ones (see below). In contrast, ΔH1 was rarely spherical and exhibited rod-like oligomers predominantly (Figure 7A and Supplementary Figure 7). This was somewhat surprising, because ΔH1 was reported to form small oligomers based on the result of size exclusion chromatography (Otters et al., 2013). The V11E mutant contained both spherical and rods (Figure 7A,B), and we detected two types of the spherical structures, namely particles (with a smooth surface) or rings (with a hole-like low density part); about 60% consisted of particles that resembled wild-type VIPP1, whereas the rest had a hole-like low-density region at the center (Figure 7C, yellow arrows) and may represent the ring structure. Similar to V11E, N16I also contained both spherical and rod-like structures. The size of spherical structures in N16I was close to the wild-type, but their shape was frequently distorted (Figure 7C). Moreover, the rod-like structures appeared to be different from those of wild-type, ΔH1, and V11E (Supplementary Figure 7).

The rod-like structures of wild-type, ΔH1, and V11E seemed to possess well organized shape with length and width that ranged from 120 to 300 nm and from 28 to 34 nm, respectively. The width was almost uniform from one end to the other side of each structure. Additionally, every structure of ΔH1 and V11E appeared to possess a low-density part at the center along the long axis (Supplementary Figure 7). Unlike the organized structure of these species, the shape of rod-like structures of N16I appeared disorganized and bumpy. Although some of them were 100–300 nm in length, huge structures were also occasionally observed (Figure 7A, red arrows). Moreover, the width was variable even within a single structure (Supplementary Figure 7). The E126Q/E179Q double mutant contained mostly spherical structures and rarely showed rod-like shapes. The shape of spherical structures was similar to the wild-type, but the size tended to be larger (see below).

We estimated the ratio between spherical and rod-like structures in wild-type and each mutant protein in accordance with Gupta et al. (2021) (Supplementary Figure 8A). As shown in Figure 7B, approximately 80% of the wild-type protein had a spherical structure, whereas ΔH1 was predominantly rod-like (∼90%). V11E had a higher ratio of rod-like structures compared with the wild-type protein. In contrast, N16I exhibited both spherical and rod-like structures at equal rates (∼50%), and E126Q/E179Q contained >90% spherical structures.

We next analyzed the size of the spherical structures. We manually measured the size of >100 spherical particles (Supplementary Figure 8B) in each protein preparation and analyzed their distribution. As shown in Figure 7D, the wild-type protein contained structures ranging from 30 to 50 nm as the majority. V11E appeared to have smaller particles than the wild-type. N16I also showed a similar tendency. On the other hand, E126Q/E179Q showed a wide range of sizes and tended to contain more spherical ones that were larger than the wild-type. Overall, the ATP/GTP hydrolysis activity appeared to correlate with both size and occupancy of spherical structure.

## Discussion

In the present study, we extended the previous analysis using AtVIPP1-His and found that AtVIPP1 has not only GTP- but also ATP-hydrolytic activity. Furthermore, this activity was confirmed using both malachite assay and HPLC analysis, which respectively detected Pi and GDP/ADP. The stoichiometric production of Pi and GDP/ADP in every reaction led us to conclude that the nucleotide hydrolysis activity of AtVIPP1 is a reaction that hydrolyzes purine triphosphate into diphosphate. Given that bacterial PspA had similar activity to VIPP1 in our previous study (Ohnishi et al., 2018), this nucleotide hydrolysis activity is likely to be a common property of VIPP1/PspA family proteins.

Vesicle-inducing protein in plastid 1 has no authentic nucleotide binding domains, so the question arises where the ATP/GTP-hydrolytic activity is located in the oligomers. Although limited to cyanobacterial VIPP1 (also termed IM30), recent cryo-EM particle analyses give some hints on the coordination of nucleotide binding in the VIPP1 oligomer (Gupta et al., 2021). In the ring structure resolved *in vitro*, the VIPP1 monomer forms a hairpin structure with H2 and H3, connected by the amphipathic N-terminal H1 helix vertically and downward coordinated in the center. At the C-terminus, the H4/H5 helix is aligned with H3 horizontally, connected by the flex amino acid stretch. H6 is twisted slightly upward against the aligned H4/H5, and its angle varies due to the flex region that connects H3 and H4/H5. H7 was not accommodated in the structure, likely due to its disordered nature. The monomer is weaved into a ring structure (C14-C18) which is further inter-weaved and assembles into a layered stack, similar to a fruit basket. H1, aligned at the luminal side, forms a hydrophobic pillar that bind lipids. In this structure, a novel nucleotide binding pocket coordinated by three monomers has been identified. Mutagenesis of the amino acid residues surrounding this pocket, E126 and E179 to glutamine (E126Q/E179Q), completely abolished GTPase/ATPase activity (Gupta et al., 2021). Based on this finding in SynVIPP1, we also created corresponding mutations in AtVIPP1. However, most of the mutations had little or no effect on its nucleotide hydrolysis activity, and somewhat surprisingly E126Q/E179Q had an incremental effect. Therefore, although the precise reason should be investigated in the future work, we considered the possibility that the GTPase/ATPase activity described here might not be attributed to the proposed cyanobacterial pocket. Despite their similarity in amino acids, the fine structure of AtVIPP1 oligomer may be different from that of SynVIPP1, because not only mutations on the nucleotide-binding pocket but also V10E differently affected them. Our negative staining EM analysis also indicated that AtVIPP1 forms oligomeric arrangements different from SynVIPP1 (see below). Since several mutations in H1 (V11E and N16I) had a negative effect on the hydrolysis activity, it is possible that H1 might play a role not only in lipid binding but also in the NTPase activity.

It has been shown that all VIPP1-related proteins form similar oligomers. The cyanobacterial VIPP1 oligomers show a basket-like structure consisting of stacked rings, whereas the oligomers of PspA and ESCRT-III are in a form more like a spiral tube (Nguyen et al., 2020; Junglas et al., 2021). Correlative light-EM tomographic analyses showed a rod-like structure of *Chlamydomonas* VIPP1 *in vivo* (Gupta et al., 2021), which resembled the structure observed *in vitro* (Theis et al., 2019). As for AtVIPP1, the fine structure has not been resolved, but large homo-oligomers were shown to be rather particle-like than ring structures *in vitro*, according to negative staining. Supporting this, the present study found that most of the wild-type and mutated AtVIPP1s were shown to form rods and particles, with the spherical particles being dominant in the wild type (∼80%). It is noteworthy that mutations such as ΔH1 reversed this ratio, forming rod-like stacking predominantly. Likewise, V11E and N16I mutants, which decreased NTPase activity, showed the tendency to accumulate more rods, although their morphologies appeared to differ slightly, both in the length of rods and the size of particles. The E126Q/E179Q mutant formed larger structures than the wild-type AtVIPP1, whereas V11E showed small particles or rod-like structures. Based on these observations, spherical structures may include those conferring high ATP/GTP hydrolysis activity. We inferred strongly that the oligomeric properties of VIPP1 differed considerably among species, because H1 is dispensable for SynVIPP1 (Thurotte and Schneider, 2019; Junglas et al., 2020), while the corresponding N-terminal region is vital to oligomerization of AtVIPP1 (Otters et al., 2013; Ohnishi et al., 2018). *In vivo*, AtVIPP1 fused to C-terminal GFP was shown to exhibit dynamic behavior of particles, which underscored the characteristic response of AtVIPP1 to stress in chloroplasts. Detailed analysis of VIPP1 oligomers, e.g., those showing homogenous ring-like structures such as V11E, may help to decipher the fine structure of AtVIPP1.

The present data demonstrate that both ATP and GTP can be converted to purine dinucleotides in AtVIPP1, which may correlate with its oligomeric assembly as evidenced by SynVIPP1. Furthermore, careful observation of these activities, in terms of dependency on pH and divalent cations as a cofactor, indicated that the ATP and GTP hydrolysis activities are biochemically distinguishable. Whereas the ATP hydrolysis activity was optimized for alkaline conditions in the presence of Ca^2+^, GTP hydrolysis was optimized at neutral pH only in the presence of Mg^2+^. Given their theoretical *V*_max_, the rate of GTP hydrolysis is 6-7 times higher than ATP hydrolysis. However, the affinity for ATP in the presence of Ca^2+^ at pH 8.5 was 4-5 times higher than that for GTP in the presence of Mg^2+^ at pH 7.5. Therefore, ATP hydrolysis with Ca^2+^ may proceed even in the presence of low concentrations of the substrate. Overall, both AtVIPP1 and SynVIPP1 share ATP/GTP hydrolysis activity, but their properties seem different. For example, SynVIPP1 has the ATP hydrolyzing rate that is almost the same as that for GTP hydrolysis (Siebenaller et al., 2021), suggesting that GTP or ATP hydrolysis reactions could overlap with each other in *Synechocystis* cells unless the concentrations of their substrates are substantially different from each other. On the other hand, the *V*_max_ of the ATP hydrolysis activity of AtVIPP1 is about half of that for GTP in the presence of Mg^2+^ at neutral pH. According to the analysis of *K*_M_, the affinity for ATP was also lower than that for GTP at pH 7.5. Therefore, the GTP hydrolysis reaction should be dominant in neutral pH conditions, provided that the concentrations of the substrates are the same or close to each other. In addition, the availability of divalent cations may influence their activities. As reported previously, cyanobacterial VIPP1 does not require any divalent cations for its GTP hydrolysis activity (Junglas et al., 2020). By contrast, the GTP hydrolysis activity of AtVIPP1 was highly dependent on Mg^2+^, and both Mg^2+^ and Ca^2+^ ions were utilized as cofactors for ATP hydrolysis of AtVIPP1. The nucleotide hydrolysis activity of AtVIPP1 may be affected by its environment more than SynVIPP1, implying proper use of these two kinds of reactions. It is possible that AtVIPP1 has established elaborate nucleotide hydrolysis associated with the development of complex membrane structures through evolution, but further study is necessary for structural validation.

A question remains whether the nucleotide hydrolysis activity is required for VIPP1 oligomer formation. Following the cyanobacterial structural analysis, the observation that disruption of the nucleotide-binding pocket correlates well with the loss of oligomeric rings demonstrated that ring formation is required for nucleotide hydrolysis (Gupta et al., 2021). However, recent work from Schneider’s group reported that oligomer formation could occur even in the absence of ATP and GTP (Siebenaller et al., 2021). Given the size of the amylose resin used in Liu et al. (2021) and the central hole of the VIPP1 ring, it is likely that the basket-like oligomer of *Nostoc* VIPP1 was formed during the purification procedure without the supply of ATP/GTP, but not in *E. coli* cells because VIPP1 was tagged with maltose-binding protein at the *N*-terminus. Therefore, the two kinds of nucleotide hydrolysis activity are probably not essential for oligomer formation, but they might make contribution, such as an increasing the efficiency of the process. *In vitro* analyses using liposomes showed that the shape of liposomes can be modified or the liposomes increased in size compared with untreated ones with the addition of SynVIPP1 even in the absence of nucleotide triphosphate (Saur et al., 2017; Junglas et al., 2020; Liu et al., 2021). Thus, the nucleotide hydrolysis reactions are not essential for membrane fusion processes in principle. However, GTP increased the initial rate of membrane fusion reaction approximately twice compared with control experiments, whereas the addition of GDP showed the opposite effect (Junglas et al., 2020). Therefore, the GTP hydrolysis reaction may increase the efficiency of membrane fusion and/or further processes. Although these nucleotide hydrolysis activities do not appear to be essential for oligomer formation *in vitro*, it might be critical for an unknown function *in vivo*, especially in physiologically adverse conditions.

In conclusion, we provide convincing evidence that AtVIPP1 has both ATP and GTP hydrolytic activities, which are mutually distinguishable at a biochemical level. The possession of these activities, although not fully resolved in terms of structure and physiological function, underscores VIPP1 as a novel remodeling molecule in the ESCRT-III/PspA/VIPP1 protein superfamily.

## Data Availability Statement

The raw data supporting the conclusions of this article will be made available by the authors, without undue reservation.

## Author Contributions

WS conceived the original research plans. NO, MS, HK, LZ, and K-IS conducted experiments. NO performed most experiments on VIPP1 purification and NTPase activity, with support from MS on HPLC analysis, HK on negative stain and EM analysis, and LZ on nucleotide binding assay. WS supervised all experiments. NO, MS, and HK analyzed data. NO and WS wrote the manuscript on behalf of all authors. All authors contributed to the article and approved the submitted version.

## Conflict of Interest

The authors declare that the research was conducted in the absence of any commercial or financial relationships that could be construed as a potential conflict of interest.

## Publisher’s Note

All claims expressed in this article are solely those of the authors and do not necessarily represent those of their affiliated organizations, or those of the publisher, the editors and the reviewers. Any product that may be evaluated in this article, or claim that may be made by its manufacturer, is not guaranteed or endorsed by the publisher.
