# Supplementary material for: Distinctive in vitro ATP Hydrolysis Activity of AtVIPP1, a Chloroplastic ESCRT-III Superfamily Protein in Arabidopsis
Source: Front Plant Sci. 2022 Jul 12;13:949578. doi: 10.3389/fpls.2022.949578 (PMC9315428; doi:10.3389/fpls.2022.949578)
Supplement: Supplementary file 1 [file Data_Sheet_1.PDF]

Supplementary Material

Contents

Supplementary Tables

Supplementary Table 1. Primers used for mutagenesis 2

Supplementary Figures

Supplementary Figure 1.	Preparation of recombinant VIPP1-His proteins.	3
Supplementary Figure 2.	Substrate specificity of the hydrolysis reaction of VIPP1 protein.	4
Supplementary Figure 3.	Representative HPLC elution profiles of authentic nucleotides (A) and reaction mixtures of the ΔH1 mutant (B)	5
Supplementary Figure 4.	Representative elution profiles of reaction mixtures of ATP- and GTP-hydrolysis reactions of WT in the presence of Ca^{2+}.	6
Supplementary Figure 5.	Mutations in the helix-1 that reduce the ATP/GTP hydrolysis activity of SynVIPP1	7
Supplementary Figure 6.	Evaluation of oligomerization state of wild-type and mutated VIPP1-His proteins.	8
Supplementary Figure 7.	Electron microscopic analysis of the rod-like structures of AtVIPP1-His	9
Supplementary Figure 8.	Estimation of the distribution of spherical and rod-like structures (A) and the size of spherical structures (B)	10

Supplementary Table 1. Primers used for mutagenesis

Type of mutation	Primer ID	sequence (5' to 3')
V10E	V10E-fw	TCTAGAGaGGTCAAGTCATATGCAAAT
	V10E-rv	CTTGACCtCTCTAGAAAATCGTTCAAA
V11E	V11E-fw	AGAGTGGaaAAGTCATATGCAAATGCG
	V11E-rv	TGACTTttCCACTCTAGAAAATCGTTC
R44K	R44K-fw	AAGATGaagCAAGCCACTGCACAGGTT
	R44K-rv	GGCTTGcttCATCTTTGTCAAATCACT
E126Q	E126Q-fw	CTCTTGcAGAGTAAGATACAAGAGGCA
	E126Q-rv	CTTACTCTgCAAGAGCCTTGTATTTGA
E179Q	E179Q-fw	GCTATGcAGTCTGAAGCAGATGCACTA
	E179Q-rv	TTCAGACTgCATAGCCATAACTTTCTC

The codons encoding mutated amino acid residues are underlined.

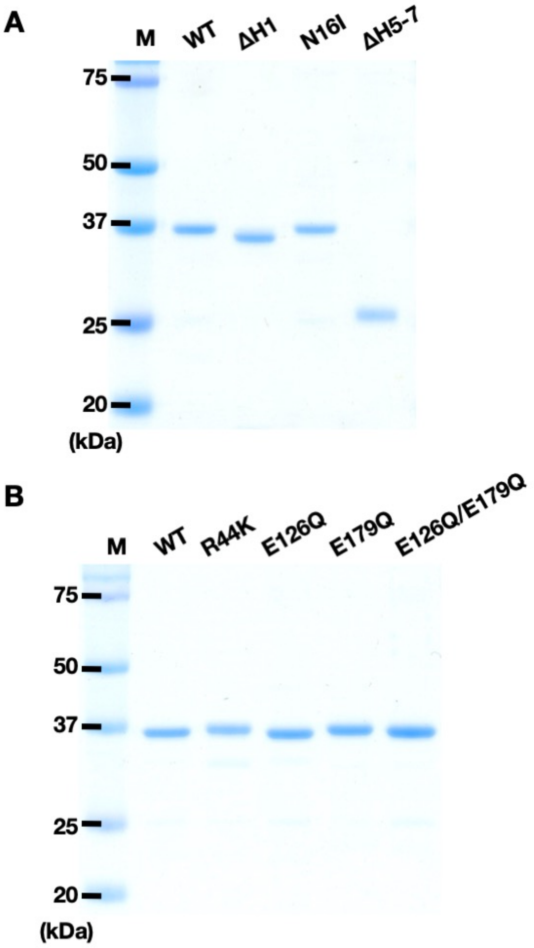

Supplementary Figure 1. Preparation of recombinant VIPP1-His proteins.

The final protein preparation (0.2 μg protein/lane) was subjected to SDS-PAGE and followed by CBB staining, confirming the majority of VIPP1-His protein in each preparation. The proteins were used in either Figure 1 (A) or Figure 5 (B).

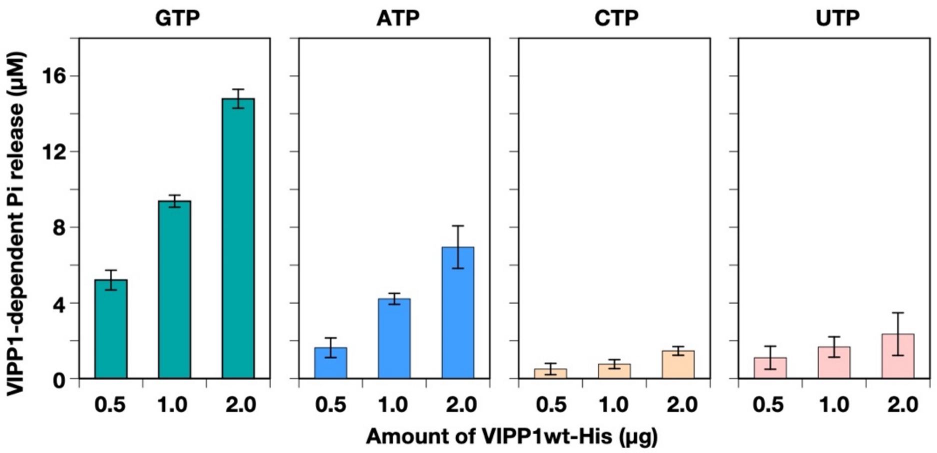

Supplementary Figure 2. Substrate specificity of the hydrolysis reaction of VIPP1 protein. The level of released Pi was analyzed with three kinds of quantity of wild-type VIPP1 protein (0.5–2.0 μg in 200 μL reaction mixture) in the presence of four kinds of nucleotide triphosphate (GTP, ATP, CTP, and UTP) as the substrate. Each bar graph and error bar represents the means and SE, respectively, of results from 4–6 independent experiments.

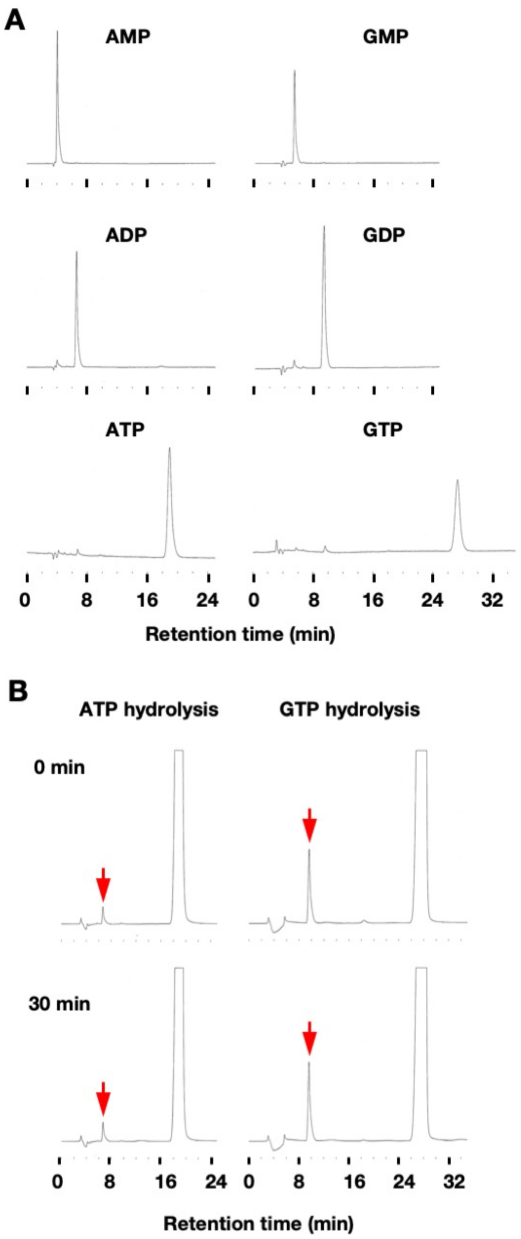

Supplementary Figure 3. Representative HPLC elution profiles of authentic nucleotides (A) and reaction mixtures of ΔH1 mutant (B).

(A) Elution profiles of solutions of authentic AMP, ADP, ATP, GMP, GDP, and GTP. (B) Representative elution profiles obtained from the ATP- and GTP-hydrolysis reactions with the ΔH1 mutant protein before (0 min) and after (30 min) incubation at 37°C. The elution peaks corresponding to ADP (column for ATP hydrolysis) and GDP (column for GTP hydrolysis) are indicated with red arrows.

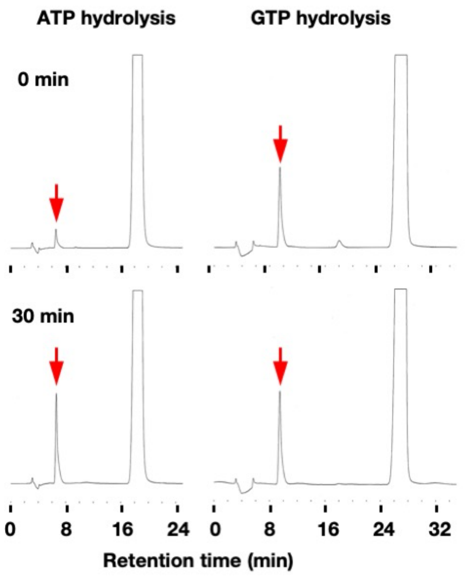

Supplementary Figure 4. Representative elution profiles of reaction mixtures of ATP- and GTP-hydrolysis reactions of WT in the presence of Ca^{2+}.

The reactions were carried out by addition of 2.5 μg VIPP1-His protein to obtain high enough signals to detect.

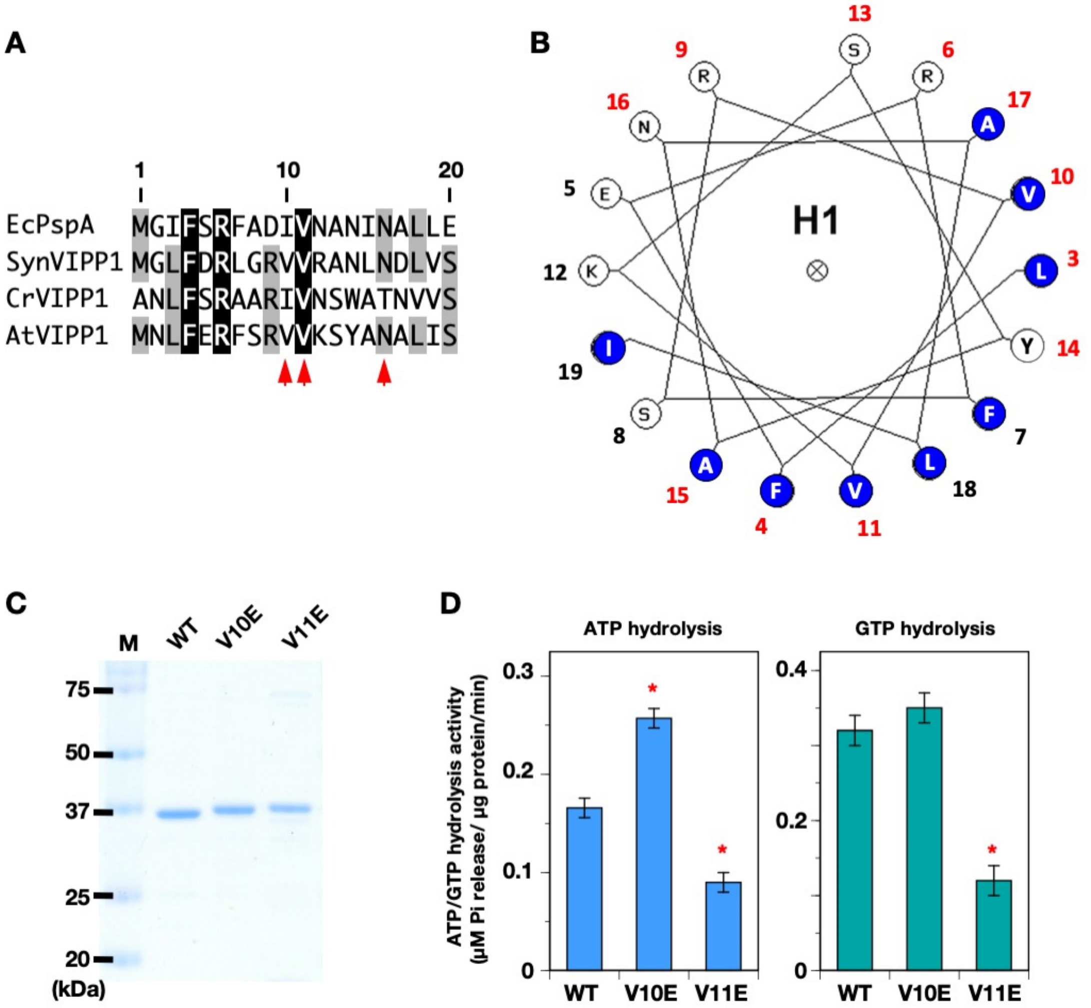

Supplementary Figure 5. Mutations in the helix-1 that reduce the ATP/GTP hydrolysis activity of SynVIPP1.

(A) Multiple alignment of sequence corresponding to the helix-1 among PspA of *Escherichia coli* and VIPP1s from three photosynthetic organisms (*Synechocystis* sp. PCC6803, *Chlamydomonas reinhardtii*, and *Arabidopsis thaliana*). Red arrows indicate the amino acid residues that are mutated in the present study (V10, V11, N16). (B) Helical wheel projection of the N-terminal region of VIPP1 from Leu3 to Ile19 (adapted from Zhang and Sakamoto (2015)). Hydrophilic and hydrophobic amino acid residues are shown in white and blue, respectively. The numbers represent positions from the N-terminus, of which residues highly conserved through species (Ohnishi et al (2018)) are red-colored. (C) SDS-PAGE for the final preparation of VIPP1s used for the hydrolysis reaction analyses in panel C, confirming the majority of the desired recombinant protein was in solution. (D) ATP- and GTP-hydrolysis activity of wild-type and two point-mutated proteins. Asterisks indicate significant differences from the values of wild-type, which was analyzed using Welch's t-test (p<0.01).

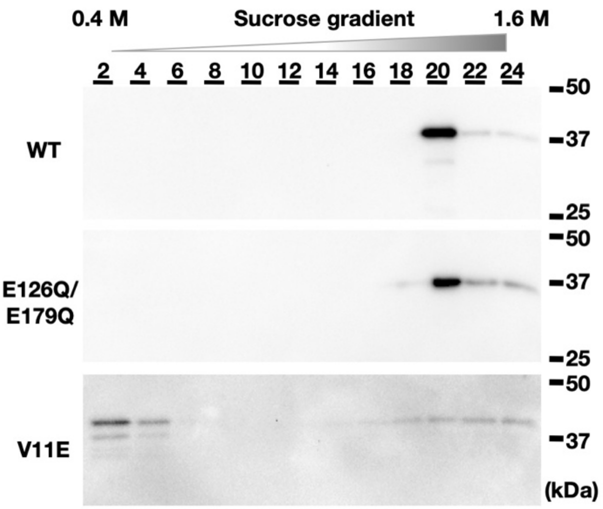

Supplementary Figure 6. Evaluation of oligomerization state of wild-type and mutated VIPP1-His proteins.

The size and density of VIPP1 oligomers were analyzed using linear sucrose density gradient centrifugation (0.4–1.6 M) and following immunoblot analyses. Among the obtained 25 fractions, those with an even number were subjected to immunoblotting analyses with the specific antibody against VIPP1. The blots are representative ones from three independent experiments.

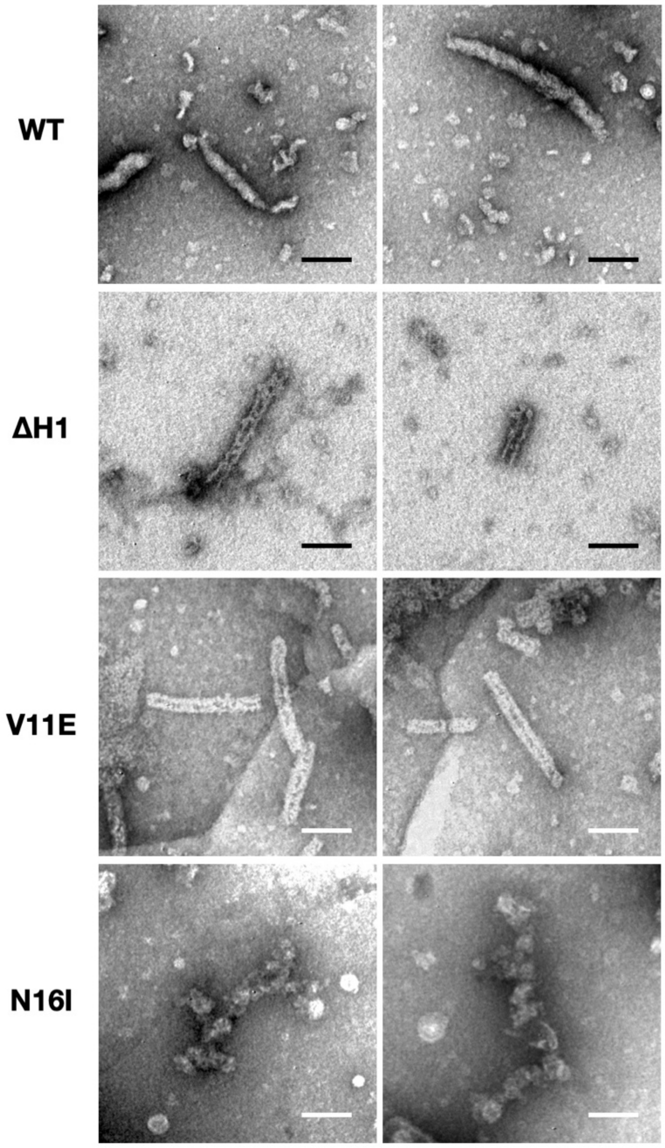

Supplementary Figure 7. Electron microscopic analysis of the rod-like structures of AtVIPP1-His.

Representative rod-like structures observed in WT, ΔH1, V11E, and N16I protein preparations. Scale bars represent 100 nm.

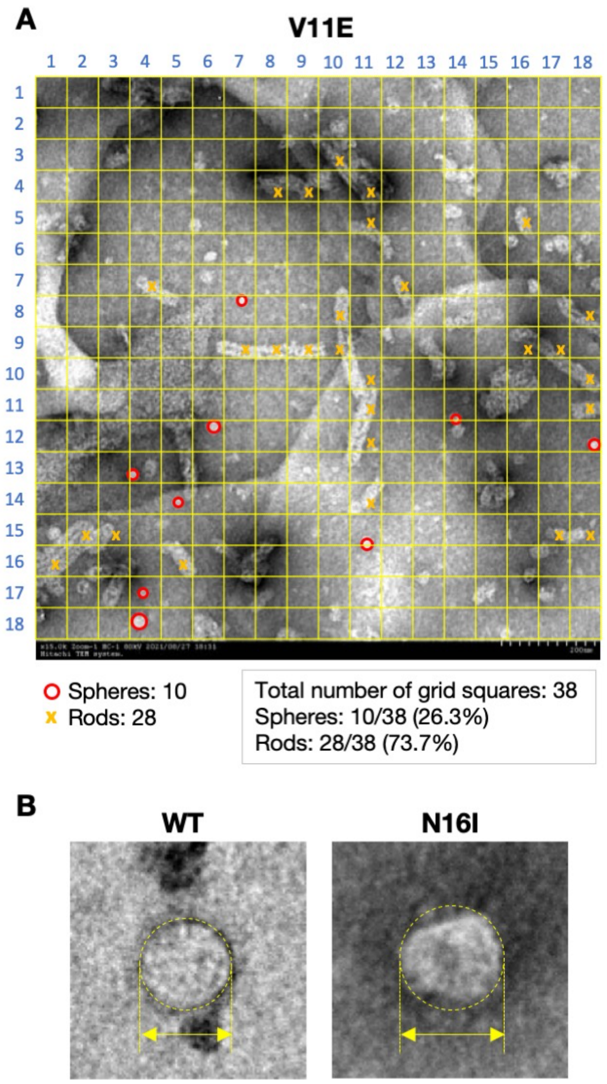

Supplementary Figure 8. Estimation of the distribution of spherical and rod-like structures (A) and the size of spherical structures (B).

(A) An example of estimation of the ratio of spherical structures to rods for the recombinant proteins based on electron micrographs. Each picture was partitioned into 324 grid squares (18 × 18). The squares occupied by spherical structures (marked with circles) or rods (marked with crosses) with more than approximately a quarter of each area were counted. (B) Estimation of the size of spherical structures. First, every spherical structure was overlaid with a perfect circle. If a structure was distorted (*e.g.* N16I shown in the right panel), the circle was minimized as long as it covered whole the structure. The diameter of the circles (shown as left to right arrows) was defined as the "size" of the spherical structures.
